# Testing Practices for Fungal Respiratory Infections and SARS-CoV-2 among Infectious Disease Specialists, United States

**DOI:** 10.3390/jof7080605

**Published:** 2021-07-27

**Authors:** Kaitlin Benedict, Samantha Williams, Susan E. Beekmann, Philip M. Polgreen, Brendan R. Jackson, Mitsuru Toda

**Affiliations:** 1 Mycotic Diseases Branch, Centers for Disease Control and Prevention, Atlanta, GA 30329, USA; pog3@cdc.gov (S.W.); iyn0@cdc.gov (B.R.J.); nrk7@cdc.gov (M.T.); 2 Carver College of Medicine, University of Iowa, Iowa City, IA 52242, USA; susan-beekmann@uiowa.edu (S.E.B.); philip-polgreen@uiowa.edu (P.M.P.)

**Keywords:** blastomycosis, coccidioidomycosis, cryptococcosis, histoplasmosis, SARS-CoV-2, COVID-19

## Abstract

In an online poll, 174 infectious disease physicians reported that testing frequencies for coccidioidomycosis, histoplasmosis, blastomycosis, and cryptococcosis were similar before and during the COVID-19 pandemic, indicating that these physicians remain alert for these fungal infections and were generally not concerned about the possibility of under-detection.

## 1. Introduction

Certain fungal infections can cause fever, cough, and shortness of breath, similar to other respiratory illnesses including COVID-19, making them challenging to diagnose [1]. Co-infection with fungi and SARS-CoV-2 is also a growing concern, particularly for COVID-19-associated pulmonary aspergillosis (CAPA) and mucormycosis [2,3]. The relationship between COVID-19 and other fungal respiratory infections is less clear, with only a few published case reports of coccidiomycosis, histoplasmosis, and cryptococcosis in COVID-19 patients [4,5,6,7,8,9,10].

In general, testing for fungal respiratory infections appears to be low among primary care providers, who are often the first point of care for patients with these diseases [11]. Consequently, these patients can experience long delays before being correctly diagnosed by specialists such as infectious disease (ID) physicians. However, little is known about fungal respiratory infection testing practices among this provider population, both generally and in the context of COVID-19. To inform strategies to reduce potential delayed and missed fungal diagnoses, we surveyed ID physicians to understand how the COVID-19 pandemic may have influenced their testing practices for coccidioidomycosis, histoplasmosis, blastomycosis, and cryptococcosis.

## 2. Materials and Methods

The Emerging Infections Network (EIN) is a provider-based surveillance network supported by CDC and IDSA [12]. EIN emailed a link to a 7-question poll three times during January–February 2021 to its >2700 member listserv (https://ein.idsociety.org/), which includes the target audience of ~1500 ID physicians who see adult patients, pediatric ID physicians, and to other healthcare and public health professionals. 

## 3. Results

In total, 199 people responded; 6 were not practicing ID physicians and were excluded. We further limited the analysis to the 174 (90%) ID physicians who had consulted on or treated any suspected or known COVID-19 patients (median 50 patients, interquartile range 20–200, range 3–2000). The highest proportion of respondents was from the West (*n* = 55, 32%), followed by the Midwest (*n* = 47, 27%), South (*n* = 46, 26%), and Northeast (*n* = 26, 15%). 

**Figure 1:**
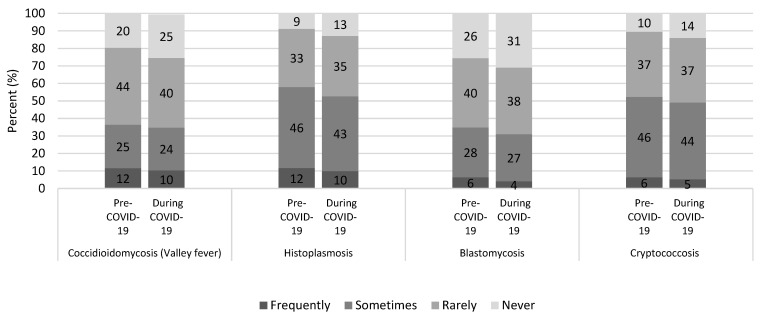
Infectious disease physicians’ reported testing frequency for coccidioidomycosis, histoplasmosis, blastomycosis, and cryptococcosis among patients with signs and symptoms of community-acquired pneumonia, before and during the COVID-19 pandemic, United States.

The proportion of respondents who reported “sometimes” or “frequently” testing for fungal infections among patients with signs and symptoms of community-acquired pneumonia before the COVID-19 pandemic was similar to the proportion that reported doing so at the time of the survey for all four infections we surveyed: coccidioidomycosis (36% vs. 35%), histoplasmosis (58% vs. 53%), blastomycosis (35% vs. 31%), and cryptococcosis (52% vs. 49%) (Figure 1). In areas where each infection is typically most common, these proportions were the same before the pandemic vs. at the time of the survey for coccidioidomycosis (83% vs. 83%) in Arizona, California, New Mexico, Nevada, Texas, and Utah combined; but they were higher before the pandemic vs. at the time of the survey for histoplasmosis (85% vs. 70%) in the Midwest and for blastomycosis (65% vs. 53%) in the Midwest [13].

Overall, most respondents (*n* = 124, 72%) said that their testing practices for fungal infections had not changed since the pandemic began. Among the 9% (*n* = 15) who said they test for fungal infections less frequently since the pandemic began, the main reasons, captured as free-text responses, were that COVID-19 was believed to be a more likely illness and reduced patient travel to regions where fungal infections are most common. Among the 20% (*n* = 34) who said they test for fungal infections more frequently since the pandemic began, common reasons included: immunosuppression and steroid use in COVID-19 patients, suspicion for CAPA, and specific concern for patients with long hospitalizations or failure to improve.

**Figure 2:**
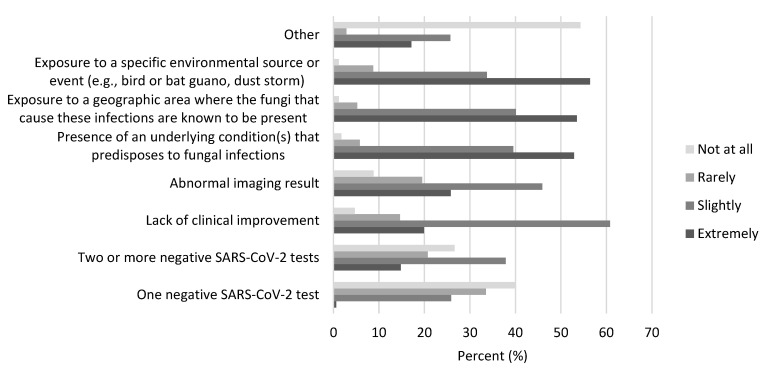
Infectious diseases physicians’ responses to the question “When considering a patient with possible COVID-19, how would each of the following influence your decision to test for coccidioidomycosis, histoplasmosis, blastomycosis or cryptococcosis?”.

The most influential factors in deciding to test possible COVID-19 patients for coccidioidomycosis, histoplasmosis, blastomycosis, and cryptococcosis included: exposure to a specific environmental source (*n* = 97, 56%), exposure to known endemic areas (*n* = 92, 53%), and presence of underlying condition(s) that predispose people to fungal infections (*n* = 91, 53%) (Figure 2). Factors less commonly noted as extremely influential were: abnormal imaging results (*n* = 41, 26%), lack of clinical improvement (*n* = 34, 20%), and two or more negative SARS-CoV-2 tests (*n* = 25, 15%). Most respondents (*n* = 94, 71%) were not concerned about underdiagnosis of these 4 fungal infections either at their institution or in general. The main reasons for lack of concern, captured as free-text responses, included: that patients are being appropriately tested for these infections, that these infections are not present in their geographic area or the patient population they care for, and greater concern for CAPA than these infections.

## 4. Discussion

ID physicians reported similar rates of testing for coccidioidomycosis, histoplasmosis, blastomycosis, and cryptococcosis before and during the COVID-19 pandemic, relying primarily on epidemiologic exposures and host status to guide testing decisions. These results indicate that respondents have continued to consider these fungal infections as a cause of respiratory illness during the COVID-19 pandemic.

Our finding that most respondents were not concerned about underdiagnosis of coccidioidomycosis, histoplasmosis, blastomycosis, and cryptococcosis in the context of COVID-19 may be justified by the existence of only a few case reports of such co-infections: 4 histoplasmosis cases in Latin America [5,9,10,14], 2 coccidioidomycosis cases in a highly endemic area of California [4,8], 2 cryptococcosis cases in immunocompromised patients [6,7], and no published reports of blastomycosis. Together with the moderate-to-high testing frequencies (depending on the disease and geographic area) in this survey, this suggests that the overlap between these fungal infections and COVID-19 may not be widely overlooked by respondents. However, delayed diagnosis of these fungal infections because of clinical similarities to other respiratory infections is a potentially larger issue than co-infections. ID physicians may be more likely to see patients with fungal infections after failed treatment for other suspected infections and are therefore probably more inclined to test for fungal etiologies than other healthcare providers. Nevertheless, overwhelming evidence indicates that these 4 fungal infections are under-detected in general, apart from COVID-19 [15]. 

In contrast to the few case reports of coccidioidomycosis, histoplasmosis, blastomycosis, and cryptococcosis associated with COVID-19, nearly 200 cases of CAPA have been described globally [16], and our results suggest that respondents were familiar with this emerging phenomenon, despite the survey’s intended focus on other infections. For example, our finding that 20% of respondents said they test for fungal infections overall more frequently now compared with pre-COVID-19 seems to contrast with the finding that testing rates were similar by time period for the 4 infections we asked about. However, this discrepancy appears to be attributable to providers including aspergillosis in their answers about overall testing. 

Our results show that respondents rely more frequently on epidemiologic exposures, geography, and host factors, rather than negative SARS-CoV-2 testing or lack of clinical improvement, to guide testing decisions for certain fungal infections. Although the survey did not ask about factors influencing testing decisions for patients without COVID-19, it is critical for clinicians to remember when considering testing that many patients with these fungal infections do not have clear epidemiologic exposures, that the regions where these infections can occur are wider than are often appreciated, and that severe disease can occur among people without underlying conditions [17]. 

Our results are based on a convenience sample and might not reflect the experiences and opinions of all U.S. ID physicians. Much remains to be learned about coccidioidomycosis, histoplasmosis, blastomycosis, and cryptococcosis and their relationship to COVID-19. It is important for clinicians to continue considering fungal pneumonias as a possible cause of respiratory illness during the COVID-19 pandemic, to reduce diagnostic delays and prevent severe outcomes.
